# (1*H*-Imidazol-4-yl)methanol

**DOI:** 10.1107/S160053681301636X

**Published:** 2013-06-26

**Authors:** Marisa B. Sanders, John C. Farrokh, Joseph Hardie, Benny C. Chan

**Affiliations:** a Department of Chemistry, The College of New Jersey, 2000 Pennington Rd, Ewing, NJ 08628, USA

## Abstract

The title compound, C_4_H_6_N_2_O, displays two predominant hydrogen-bonding inter­actions in the crystal structure. The first is between the unprotonated imidazole N atom of one mol­ecule and the hy­droxy H atom of an adjacent mol­ecule. The second is between the hy­droxy O atom of one mol­ecule and the imidazole N—H group of a corresponding mol­ecule. These inter­actions lead to the formation of a two-dimnensional network parallel to (10-1). C—H⋯O inter­actions also occur.

## Related literature

For background information on imidazole complex formation, see: Bauman & Wang (1964 ▶); Fan *et al.* (2000 ▶). For related structures, see: Nyamori *et al.* (2010 ▶); Albov *et al.* (2006 ▶). For the use of imidazole-containing compounds in coordination chemistry, see: Huff *et al.* (1993 ▶); Fujita *et al.* (1994 ▶). For the use of the title compound in the synthesis of biological compounds, see: Darby *et al.* (1942 ▶).
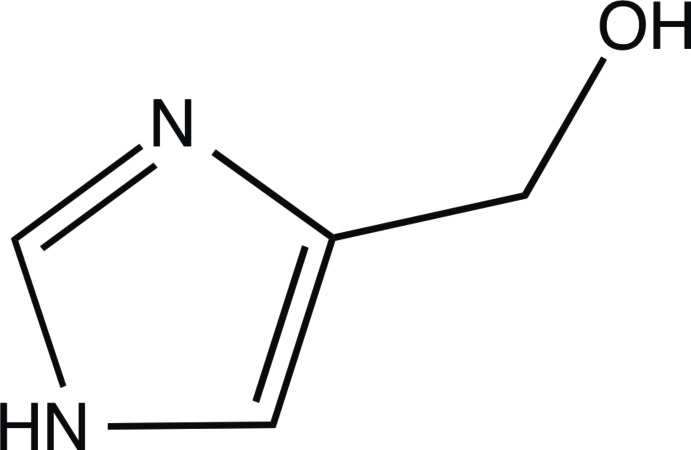


## Experimental

### 

#### Crystal data

C_4_H_6_N_2_O
*M* *_r_* = 98.11
Monoclinic, 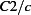

*a* = 13.9180 (9) Å
*b* = 7.1980 (5) Å
*c* = 11.6509 (12) Å
β = 125.249 (1)°
*V* = 953.20 (13) Å^3^
*Z* = 8
Mo *K*α radiation
μ = 0.10 mm^−1^
*T* = 100 K
0.52 × 0.37 × 0.29 mm

#### Data collection

Bruker APEXII CCD diffractometer
Absorption correction: multi-scan (*SADABS*; Bruker, 2011 ▶) *T*_min_ = 0.688, *T*_max_ = 0.746
5389 measured reflections
1158 independent reflections
1086 reflections with *I* > 2σ(*I*)
*R*_int_ = 0.015

#### Refinement

*R*[*F* ^2^ > 2σ(*F* ^2^)] = 0.035
*wR*(*F* ^2^) = 0.095
*S* = 1.07
1158 reflections
65 parameters
H-atom parameters constrained
Δρ_max_ = 0.35 e Å^−3^
Δρ_min_ = −0.27 e Å^−3^

### 

Data collection: *APEX2* (Bruker, 2011 ▶); cell refinement: *SAINT* (Bruker, 2011 ▶); data reduction: *SAINT*; program(s) used to solve structure: *SHELXS97* (Sheldrick, 2008 ▶); program(s) used to refine structure: *SHELXL97* (Sheldrick, 2008 ▶); molecular graphics: *CrystalMaker* (CrystalMaker Software, 2009 ▶); software used to prepare material for publication: *enCIFer* (Allen *et al.* 2004 ▶).

## Supplementary Material

Crystal structure: contains datablock(s) I, global. DOI: 10.1107/S160053681301636X/fj2622sup1.cif

Structure factors: contains datablock(s) I. DOI: 10.1107/S160053681301636X/fj2622Isup2.hkl

Supplementary material file. DOI: 10.1107/S160053681301636X/fj2622Isup3.cml

Additional supplementary materials:  crystallographic information; 3D view; checkCIF report

## Figures and Tables

**Table 1:** Hydrogen-bond geometry (Å, °)

*D*—H⋯*A*	*D*—H	H⋯*A*	*D*⋯*A*	*D*—H⋯*A*
O1—H1⋯N1^i^	0.84	1.92	2.7563 (13)	172
N2—H2⋯O1^ii^	0.88	1.99	2.8315 (11)	161
C4—H4⋯O1^iii^	0.95	2.57	3.4574 (17)	155

Symmetry codes: (i) 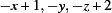; (ii) 
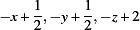
; (iii) 
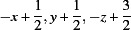
.

**Fig. 1.**
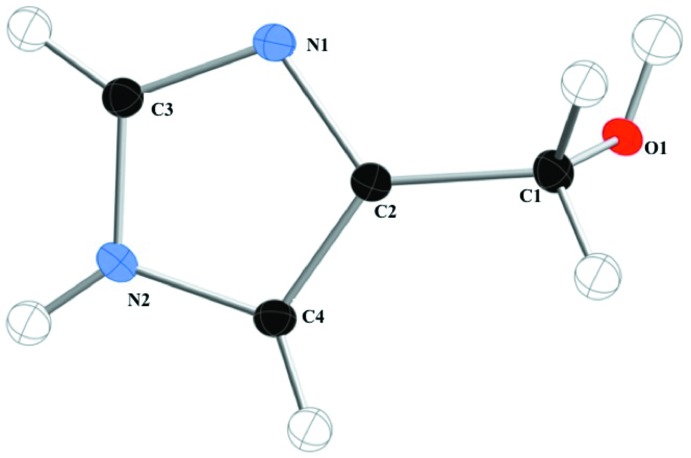
Thermal ellipsoid plot at 50% probability.

**Fig. 2.**
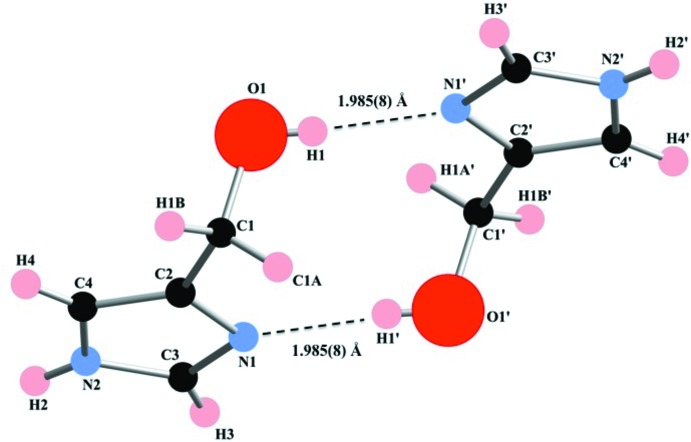
The title structure is stabilized by hydrogen bonds between N1' and H1, which each measure 1.985 (8) Å. Oxygen atoms are shown in red, carbon atoms in black, hydrogen atoms in pink, and nitrogen atoms in blue.

**Fig. 3.**
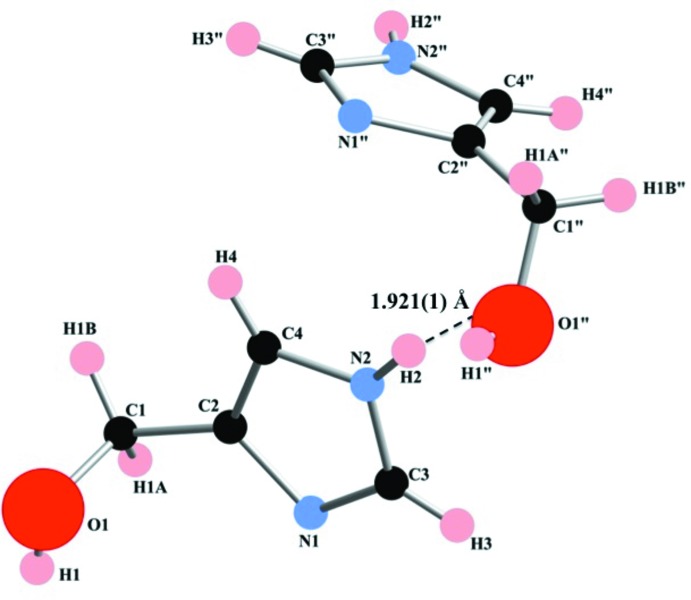
Hydrogen bonding between the oxygen (O1") on the hydroxyl group of one molecule and the hydrogen (H2) bonded to a nitrogen (N2) on the imidazole ring of a corresponding molecule measures 1.921 (1) Å.

## supplementary crystallographic information

### Comment

Imidazole ligands have been used in coordination chemistry with great success over the last twenty years (Huff *et al.*, 1993). These successes can be attributed to how the nitrogen in imidazole assists in the formation of metal complexes (Fujita *et al.*, 1994). Imidazole-containing metal complexes have a variety of applications, such as redox mediators in enzyme-based electrochemical sensors (Fan *et al.*, 2000). A few examples of imidazole complex compounds with biological applications have been reported (Bauman and Wang, 1964). Histidine, an essential amino acid, and histamine, a bioorganic compound that acts as neurotransmitter, both involve (1*H*-imidazol-5-yl)methanol in their respective synthesizes (Darby *et al.*, 1942). Here we report on a new imidazole compound, the hydroxymethyl-substituted imidazole, the title compound, C_4_H_6_N_2_O. The bond lengths and bond angles are within normal ranges in the molecular structure of this compound (Fig. 1). The compound forms hydrogen bonds of 1.985 (8) Å between the nitrogen (N1) on the imidazole ring of one molecule and the hydrogen (H1') of the hydroxyl on an adjacent molecule (Fig. 2). Hydrogen bonding also takes place between the oxygen (O1") on the hydroxyl group of one molecule and the hydrogen (H2) bonded to a nitrogen (N2) on the imidazole ring of a corresponding molecule. This bond measures 1.921 (1) Å (Fig. 3).

### Experimental

Approximately 100 mg of the target compound was dissolved in 2 ml of a 50% methanol: 50% toluene solution. The solution was allowed to evaporate slowly for two weeks until clear, colorless crystals formed. A crystal was isolated and analyzed on a Bruker *APEX* II CCD single-crystal X-ray diffractometer.

### Refinement

The structure was solved using direct methods (Bruker, 2011).

### Crystal data

**Table:**

C_4_H_6_N_2_O	*F*(000) = 416
*M**_r_* = 98.11	*D*_x_ = 1.367 Mg m^−^^3^
Monoclinic, *C*2/*c*	Mo *K*α radiation, λ = 0.71073 Å
*a* = 13.9180 (9) Å	Cell parameters from 189 reflections
*b* = 7.1980 (5) Å	θ = 3.6–28.2°
*c* = 11.6509 (12) Å	µ = 0.10 mm^−^^1^
β = 125.249 (1)°	*T* = 100 K
*V* = 953.20 (13) Å^3^	Blocks, colourless
*Z* = 8	0.52 × 0.37 × 0.29 mm

### Data collection

**Table:**

Bruker APEXII CCD diffractometer	1158 independent reflections
Radiation source: fine-focus sealed tube	1086 reflections with *I* > 2σ(*I*)
Graphite monochromator	*R*_int_ = 0.015
Detector resolution: 8.3333 pixels mm^-1^	θ_max_ = 28.6°, θ_min_ = 3.4°
ω and φ scans	*h* = −18→18
Absorption correction: multi-scan (*SADABS*; Bruker, 2011)	*k* = −9→9
*T*_min_ = 0.688, *T*_max_ = 0.746	*l* = −15→15
5389 measured reflections	

### Refinement

**Table:**

Refinement on *F*^2^	Primary atom site location: structure-invariant direct methods
Least-squares matrix: full	Secondary atom site location: difference Fourier map
*R*[*F*^2^ > 2σ(*F*^2^)] = 0.035	Hydrogen site location: inferred from neighbouring sites
*wR*(*F*^2^) = 0.095	H-atom parameters constrained
*S* = 1.07	*w* = 1/[σ^2^(*F*_o_^2^) + (0.0511*P*)^2^ + 0.6749*P*] where *P* = (*F*_o_^2^ + 2*F*_c_^2^)/3
1158 reflections	(Δ/σ)_max_ = 0.002
65 parameters	Δρ_max_ = 0.35 e Å^−^^3^
0 restraints	Δρ_min_ = −0.27 e Å^−^^3^

### Special details

**Table:**

Geometry. All e.s.d.'s (except the e.s.d. in the dihedral angle between two l.s. planes) are estimated using the full covariance matrix. The cell e.s.d.'s are taken into account individually in the estimation of e.s.d.'s in distances, angles and torsion angles; correlations between e.s.d.'s in cell parameters are only used when they are defined by crystal symmetry. An approximate (isotropic) treatment of cell e.s.d.'s is used for estimating e.s.d.'s involving l.s. planes.
Refinement. Refinement of (*F*^2^) against ALL reflections. The weighted *R*-factor *wR* and goodness of fit *S* are based on *F*^2^, conventional *R*-factors *R* are based on *F*, with *F* set to zero for negative *F*^2^. The threshold expression of *F*^2^ > σ(*F*^2^) is used only for calculating *R*-factors(gt) *etc*. and is not relevant to the choice of reflections for refinement. *R*-factors based on *F*^2^ are statistically about twice as large as those based on *F*, and *R*- factors based on ALL data will be even larger.

### Fractional atomic coordinates and isotropic or equivalent isotropic displacement parameters (Å^2^)

**Table:**

	*x*	*y*	*z*	*U*_iso_*/*U*_eq_	
O1	0.39678 (6)	0.03753 (10)	1.06291 (7)	0.0178 (2)	
H1	0.4528	−0.0374	1.093	0.027*	
N1	0.43471 (7)	0.23365 (12)	0.85046 (9)	0.0176 (2)	
N2	0.27914 (7)	0.41114 (12)	0.71171 (9)	0.0178 (2)	
H2	0.2243	0.4731	0.6368	0.021*	
C1	0.43706 (9)	0.22305 (14)	1.06767 (10)	0.0189 (2)	
H1A	0.5241	0.2245	1.1246	0.023*	
H1B	0.4118	0.3067	1.113	0.023*	
C2	0.38850 (8)	0.29121 (13)	0.92295 (10)	0.0160 (2)	
C3	0.36594 (9)	0.30971 (14)	0.72413 (11)	0.0178 (2)	
H3	0.3766	0.2945	0.6512	0.021*	
C4	0.29183 (9)	0.40012 (14)	0.83752 (11)	0.0179 (2)	
H4	0.2433	0.4567	0.8605	0.021*	

### Atomic displacement parameters (Å^2^)

**Table:**

	*U*^11^	*U*^22^	*U*^33^	*U*^12^	*U*^13^	*U*^23^
O1	0.0161 (4)	0.0178 (4)	0.0184 (4)	0.0035 (3)	0.0093 (3)	0.0032 (3)
N1	0.0164 (4)	0.0173 (4)	0.0191 (4)	0.0018 (3)	0.0103 (4)	0.0007 (3)
N2	0.0165 (4)	0.0164 (4)	0.0178 (4)	0.0027 (3)	0.0083 (3)	0.0024 (3)
C1	0.0197 (5)	0.0181 (5)	0.0154 (5)	0.0009 (4)	0.0081 (4)	−0.0012 (4)
C2	0.0159 (5)	0.0140 (4)	0.0169 (5)	−0.0009 (3)	0.0088 (4)	−0.0016 (3)
C3	0.0182 (5)	0.0168 (5)	0.0193 (5)	0.0005 (4)	0.0113 (4)	0.0005 (4)
C4	0.0184 (5)	0.0165 (5)	0.0196 (5)	0.0019 (4)	0.0114 (4)	−0.0001 (4)

### Geometric parameters (Å, º)

**Table:**

O1—C1	1.4369 (12)	C1—C2	1.4917 (13)
O1—H1	0.84	C1—H1A	0.99
N1—C3	1.3251 (13)	C1—H1B	0.99
N1—C2	1.3877 (12)	C2—C4	1.3674 (14)
N2—C3	1.3459 (13)	C3—H3	0.95
N2—C4	1.3738 (13)	C4—H4	0.95
N2—H2	0.88		
			
C1—O1—H1		H1A—C1—H1B	
C3—N1—C2		C4—C2—N1	
C3—N2—C4		C4—C2—C1	
C3—N2—H2		N1—C2—C1	
C4—N2—H2		N1—C3—N2	
O1—C1—C2		N1—C3—H3	
O1—C1—H1A		N2—C3—H3	
C2—C1—H1A		C2—C4—N2	
O1—C1—H1B		C2—C4—H4	
C2—C1—H1B		N2—C4—H4	

### Hydrogen-bond geometry (Å, º)

**Table:**

*D*—H···*A*	*D*—H	H···*A*	*D*···*A*	*D*—H···*A*
O1—H1···N1^i^	0.84	1.92	2.7563 (13)	172
N2—H2···O1^ii^	0.88	1.99	2.8315 (11)	161
C4—H4···O1^iii^	0.95	2.57	3.4574 (17)	155

Symmetry codes: (i) −*x*+1, −*y*, −*z*+2; (ii) −*x*+1/2, −*y*+1/2, −*z*+2; (iii) −*x*+1/2, *y*+1/2, −*z*+3/2.
